# A Summary View of the Ætiology of Bilious Fever

**Published:** 1829-02

**Authors:** John P. Harrison

**Affiliations:** Louisville, Ky.


WESTERN JOURNAL

OF THE

MEDICAL & PHYSICAL SCIENCES.

FEBRUARY, 1829.

### ESSAYS AND CASES.

Art. I.—*A summary view of the Ætiology of Bilious Fever.* By John P. Harrison, M. D. of Louisville, Ky.

The design of the following remarks is to present a condensed statement, composed of materials, drawn from the most authentic sources, of the causes of our summer and autumnal fevers. A dark cloud of mystery hangs over many causes of disease, which is not easily penetrated by the most searching scrutiny. Causation demands much patient assiduity of research, from every one who would enter its secret recesses, where the first elements of natural phenomena are generated. Aware of the misleading attractions of a hasty generalization, by which exceptions are sometimes exalted into rules, and deviations made the standard of regular processes, I will lay down three fundamental propositions, or cardinal points, which the existing state of our professional information seems to warrant us in regarding, as a substantial groundwork upon which to erect the ætiology of bilious fever.

There are two preliminary observations to be made, more completely to open the way for the progress of the discussion. The first is, that when the word *ætiology* is used in the following pages, it is intended to import what its derivation implies. It is derived from the Greek word *aitos*, the cause of any thing; and *logos*, a discourse. Perhaps it may be regarded as a word borrowed directly from Quintillian's rhetorick,—"ætiology being a figure, by which in relating an event we assign a cause for it."

The second observation is, that in using the generic term *Bilious Fever*, all the varieties of summer and autumnal fever, which are met with in the valley of the Mississippi, from the lightest grades of ague and fever up to the all subduing and mortal power of yellow fever, are comprehended.

With these introductory remarks, the intended analysis of the remote causes of bilious fever is now entered upon.

The atmosphere by which our globe is surrounded, and in which we are immersed, is the great laboratory of nature, as regards many of those operations which have a direct reference to, and intimate connection with, the well being of man. Besides its physical qualities, such as heat and cold, dryness and moisture, alternate condensation and rarefaction, increased weight or diminished pressure, the atmosphere possesses chemical attributes, which once lay profoundly hidden from the eye of philosophy, but have been triumphantly drawn from their state of concealment by the penetrating genius of a Priestly and a Lavoisoir. But, independently of the above mentioned properties, the atmospheric fluid, which is circumfused on every hand, is the depository, or store-house for a vast variety of gaseous substances, as well as the great region in which electricity and caloric reside.

Climate comprehends these physical phenomena. To this source we are to advert for the most operative causes of common maladies. The endless variety of phlegmasial diseases is referrible to the heat and cold, humidity and aridity of the atmosphere. But there are epidemic visitations of disease, which arise irrespective and independent of these physical alterations. These must depend then, on some other change, than that of a sensible kind, in the atmosphere, or they originate from a superadded foreign ingredient infused into the air from the earth.

Hippocrates to cut the matter short, and at one blow to prostrate all opposition to his peculiar views, attributed such visitations to a certain something, about the meaning of which learned commentators are not agreed, a *to theion*;—the god; for being a Grecian, he imitated his countrymen in peopling the air with imaginary deities. Now this is a mere superstition, and deserves no further notice. Noah Webster, however, informs us, in his elaborate work on Epidemics, that Hippocrates taught the existence of a *katastasis loimodes*, or pestilential constitution, or condition of atmosphere; and Sydenham in his imperishable work does the same. But these two great cultivators of medicine merely state a fact, without tracing the occult nature of the cause. Lancisi attempted to prove, that besides the sensible qualities of the atmosphere, and the epidemic constitution, there did exist an intermediate agent, which though inappreciable by our senses, or discoverable by chemical tests, was yet a most efficient cause of fever. Marsh miasma, though acting independently of the epidemic state, or condition of the atmosphere, Lancisi considered, as in some degree sustaining a relation to the agency of heat, having its origin from a hot sun operating on stagnant water, impregnated with vegetable matter. Consonant to this short exposition, there emerge before our view, three distinct sources of disease, as far as the atmosphere has any agency in its production.

1st. The sensible qualities, or physical changes of the air—this Dr. J. M. Smith, in his work on Epidemics, calls sensible meteoration.

2d. Marsh miasma—denominated variously by the terms paludal effluvia, malaria, vegeto-animal effluvia, and exhalations from the soil.

3d. Epidemic condition of the atmosphere, termed thus by Hippocrates, and Sydenham, and called by Dr. Smith, epidemic, or insensible meteoration.

Among the most efficient agents in developing the pestiferous miasma, to which epidemic bilious fever owes its origin, is high solar heat. That high solar heat however, uncombined with some inquinated state of the air, will produce epidemic bilious fever, is a position at variance with the most ordinary observation. Locality of position, and other modifying circumstances, come in as qualifying considerations to the universality of this statement. I refer to our climate, and to the location of this city, when I speak with such confidence. And I am borne out by the concurrent testimony of other physicians, as respects the truth of the position, in reference to other places.

There are cases of fever of a sporadic character, occurring every summer, from excessive heat, but such cases are more manageable than epidemic cases, arising from causes of a more intense morbid influence. Some medical gentlemen, abandoning the ground that heat alone can produce epidemic bilious fever, have resorted to the conjoint action of solar heat and humidity, as affording a just foundation upon which to establish the ætiology of the disease. Fordyce assumed this ground, and with considerable adroitness strove to maintain it. But however ingenious may be the exposition of his peculiar views, yet in matter of fact observation, his system is extremely defective.

Let the reader reflect on the following language of the learned author:—"When the air is moist in consequence of water evaporating from a marshy country, or from canals in which the water is stagnating or moving with a very slow motion, fevers more frequently arise, than when the moisture proceeds from the sea, large lakes, or rivers confined within their banks, and running with a considerable degree of rapidity. While fevers are frequently produced in the fens of Lincolnshire, few arise on the banks of the Thames." Again Dr. Fordyce says, "Since water being applied in a mass, that is to say, if a man immerses the whole, or any part of his body in water of the temperature of the atmosphere, in which he remains for some time, or if he throws water of such heat in his stomach, no disease ensues: and since water in small particles in the atmosphere applied to the body, is of the same heat with the atmosphere, and is often applied to parts of the body only, and still produces no fever; *it cannot be the mere application of the particles of the water that produces the disease; it*

*must therefore be something that they apply to the body, which occasions it. What this may be is not very clear.*"*

Here we have an admission on the part of the ablest defender of this recently revived opinion, which amounts to an acknowledgment that the whole hypothesis is untenable.

I will adduce a few facts, and leave the reader to his own inferences.

In the summer of 1823, the ponds in the neighbourhood of this city were quite full, for the rain had fallen in copious and heavy showers early in the season. Yet the town and country adjacent were healthy.

Pringle informs us of a similar fact, as witnessed by himself in Holland. Senac records the case of a city in proximity to a lake, whose inhabitants remained healthy until the lake was drained, when a fever of a malignant character speedily followed.

Ferguson, in his luminous paper on Marsh Poison, republished in the 7th vol. of Chapman's Journal, has the following remarks:--"In some ships of our navy, the fresh water, instead of being put in casks, has been preserved in bulk, by constructing a large open tank, of tin or lead, at the bottom of the hold, without in the least affecting the health of the crew, though they slept immediately above it." He adduces other instances corroborative of his own assertion that "aqueous putrefaction" will not originate the poison termed marsh miasm.

Bancroft has accumulated a mass of documentary matter, subversive of Dr. Fordyce's theory. He refers to exhalations from the sea, and from peat-bogs, as directly disproving the hypothetical suggestion that fever is attributable to mere aqueous exhalations, or to the combined operation of heat and humidity, independent of some adscititious element. I regret the necessity of dismissing this part of my subject without an ampler illustration of it; and would most earnestly refer the reader to Ferguson and Bancroft for a more complete explication.

* Fordyce on Fever, pp. 67 and 68.

The amount of all that is recognized as veritable on this point is, that though heat is the principal agent in eliciting the noxious element of bilious fever, from the dormant state in which it lies, yet that it alone will not produce that disease.

Heat is a frequent exciting cause of bilious fever, and may produce insulated cases of that disease, apparently independent of marsh effluvia, or an epidemic constitution of the atmosphere. Excess in drinking spirituous liquors, or in eating, will do the same. The chemical qualities of the atmosphere have nothing to do with the production of epidemic fever. It has been found that in the Pontine marshes, the oxygen, hydrogen, and carbonic acid gases, bear the same relative proportion to each other, that they do on any elevated salubrious spot.

I must advance to the discussion of the second general proposition, viz: that bilious fever does originate in the majority of instances, when it assumes an epidemic character, from the presence and agency of some potential substance, or materia morbi, designated by the terms marsh poison, marsh miasm, &c. The word *miasma* in Greek signifies any thing which pollutes, or corrupts; according to which derivation, the familiar phrase marsh miasmata in the plural, is not amenable to any criticism from any hypothetical ambiguity, involved in it. What is meant by the expression marsh miasma is merely the statement of a fact, that a certain emanation of a polluting quality, owes its existence to marshes, or to impure stagnant water, acted on by a hot sun. Those who deny the existence and agency of such an emanation, suppose that they are taking high but defensible ground, from which though they make no encroachments on the field occupied by their opponents, yet they confidently rely on their entrenchments to roll back the tide of war as it threatens to level their foundations to the earth. In other words, by giving a positive denial to the existence of marsh miasma, they endeavor to throw the labor of proof and demonstration on those who contend for its reality. Now let us shift the ground, and take these non-miasmatic physicians on their own gratuitous supposition. Suppose, for what will not supposition accomplish? that there is no such substance as marsh miasma, and that the many things which have been said and written about it, are but the idle effusions of those who were misled, by an "*Imago vana, quæ porta fugiens eburna somnium ducit.*"

To such gentlemen I would address myself as follows:— Do you admit the existence and agency of contagious matter, exercising its dominion through the medium of the atmosphere? Their affirmative answer would be given, without hesitation. Take for an example measles, or hooping-cough. You allow that these are contagious diseases, communicable through an atmospheric medium. Have you ever detected the morbid element that floats through the air, which emanating from patients afflicted with either of those maladies, is capable of imparting a like disease to the systems of those who have never been shielded from such an attack by a previous visitation of the affection? And yet you contend with exceeding pertinacity, that measles and hooping-cough, are produced by undetectable and inappreciable materiæ morborum or elements of disease, which have never been smelt, seen, felt, or analysed by chemical experiments. It will be replied, that we see the disease in the living human system, and that from this source it spreads around. But do we not see the actual presence of stagnant water, operated on by a hot sun, and in a sequence undeviating as that noticed with regard to the communicability of measles and hooping-cough, does not bilious fever, in some of its forms or types, make its appearance in a consecutive order, as consequential to such a conjuncture of natural phenomena? Then the actual subsistent presence and agency of marsh miasma in the atmosphere, are as demonstrable as the presence and agency of contagious effluvia. You are incapable of communicating either measles or hooping-cough by inoculation—absolute contact is not requisite to impart either malady—there is then a something, termed contagious miasm or virus, coming from a patient laboring under either affection, which infringes on the unprotected systems of persons in health, and kindles up a like malady. The character of measles or hooping-cough, is the same in its essential features and complexional hue, all over the globe. A

prevailing epidemic may impress certain modifying influences on both of them, but never obliterates the distinguishing characteristics of either malady. So, wherever a hot sun and marshy soil are found, the type or character of the fever produced by their co-operative agency is the same—varying in *degree* but not in *nature*, according to the intensity of the solar power, and the eligible condition of the soil for the evolution of such pestiferous exhalations. Some non-miasmatic physicians, escaping from the enthralment of what they deem visionary absurdities, soar to the moon in hope of finding a rational solution of the difficulties presented by epidemic fever. But the advice of Horace will come in suitably here—

Dum vitat humum, nubes et inania captet.

Whilst he avoids the ground, he takes hold of the clouds and emptiness. To those who prefer a "visit to the moon" rather than a belief in marsh miasm, I would say, that I have neither time nor patience to attempt the flight.

A few facts must now be brought to bear on this point; and the following are altogether incapable of rational solution, unless we admit the existence of marsh miasma.

Dr. Lind states that whenever the sailors were allowed to sleep on shore, on the coast of Guinea, particularly in the Island of St. Thomas, they were certain, almost to a man, of being destroyed by fever, whilst the rest of the crew, who had not visited the shore, escaped.

Dr. J. Johnson adduces a fact of like scope, with regard to the fatal Island of Edam, in the East Indies; not a man, says he, who slept a single night on this fatal spot escaped an attack of fever, and every individual thus attacked died, except three or four, who were under the mercurial impression.

Lancisi has made similar remarks with regard to the Pontine marshes.

Dr. Rush states, that a family in Philadelphia, were afflicted with bilious fever for several summers in succession, the neighbouring families remaining healthy, until the attending physician pointed out a duck puddle in the yard, as the probable origin of the sickness, which being filled up, health was restored to the inmates of the house.

But analagous facts spring up on every part of this broad field of inquiry, and like the drops of blood from the fabled head of Medusa, which turned into serpents, our feet become entangled in the multitude of melancholy examples, which beset us on every hand.

I will give one other fact, new to the profession, communicated by a late distinguished man, of great intellectual attainments. Bishop Heber, in his travels in British India, p. 374, vol. 1, speaking of his visit to the Himalaya Mountains, says, he had to traverse a tract of woody country, of which he writes in the following terms:—"I asked Mr. Boulderson" (who appears to have been an intelligent English collector, living in this part of India) "if it were true, that the monkeys forsook these woods during the unwholesome months. He answered that not the monkeys only, but every thing which had the breath of life, instinctively deserts them, from the beginning of April to October. The tigers go up to the hills, the antelopes and wild hogs make incursions into the cultivated plain; and those persons, such as dak-bearers, or military officers, who are obliged to traverse the forest in the intervening months, agree, that not so much as a bird can be seen or heard in the frightful solitude. *Yet during the time of the heaviest rains, while the water falls in torrents, and the cloudy sky tends to prevent evaporation from the ground, the forest may be passed in tolerable safety.* It is in the extreme heat, and immediately after the rains have ceased, in May, the latter end of August, and the early part of September, that it was most deadly." The above facts, given by a pious and learned man, who had no medical theory to establish, outweigh all the subtle and argute reasonings of the anti-miasmatic physicians, from the philosophic Fordyce, down to most ordinary contributors to our medical journals.

I must decline giving any facts from my own observation—referring the reader to my paper, published in the 8th vol. of Chapman's Journal, on the epidemic fever of 1822, as it prevailed in Louisville and its vicinity, for some additional proof on the subject.

Though miasmatic exhalations have a real existence, separate from the ordinary appreciable qualities of the atmosphere, yet they are susceptible of a more rapid transmission, as well as a more concentrated application, by a union with moisture. Even the pungent and volatile aromæ of plants require some degree of humidity to diffuse themselves. The electrical fluid, though an imponderable substance, employs the aqueous particles of the atmosphere, as a medium for its transmission and more extensive agency. The fact, that the operation of miasma on the human system is aided by the presence of moisture in the atmosphere, accounts for the greater degree of danger to be apprehended after night-fall, and in humid weather.

Those persons who inhabit houses that have no cellars, or the cellars of which are filled with water, are far more liable to attacks of fever in our climate than those who live on more elevated and arid sites. Upper stories for bed chambers are, therefore, preferable to the lower, as has been witnessed frequently in the Indies.

Miasmata are transmissable only a limited distance—water absorbs them, as was proved when the British army lay at the fatal island of Walcheren; the ships anchored a little distance from shore escaped their destructive presence. Trees, or rows of buildings, arrest them, as is seen in Rome, where their operation is confined to particular streets.

The sources of marsh poison are the muddy sides of creeks and rivers, mill ponds, and other stagnant waters, which present to the sun a surface upon which the *drying process* can be established. The fact that the noxious element is eliminated during the drying process, was long since observed, though accounted for on untenable, visionary grounds. In the translation of Lancisi's work on the noxious exhalations of marshes, to be found in the 13th volume of the New-York Medical Repository, we find the following remarks:—"Among the Latins, we read that M. Varro speculated on this subject, and ascribed the mischief to swarms of insects. 'It is worthy of remark, he writes, in marshy places, *that as they dry up*, there are produced certain very small animals, too minute for observation by the eye; which being taken into the body by the mouth and nostrils, are the cause of different diseases.' "

"Palladio has expressed himself in nearly the same terms, where he says; 'A marsh is to be avoided upon every principle, especially on the south or west, or if it usually *dries up* in summer, because it generates pestilence," &c.—pp. 244–5.

However exceptionable to a sound inductive spirit of research, the hypothetical figments of these ancient generalizers may be, yet they state facts not controvertible by the cavils of any non-miasmatic physician. Here then the question may be left;—the few facts above quoted are mere drops, to the full tide of proof that rolls its broad waves along the deep channels of medical discussion; and upon which the most sceptical may, if he *will*, be quickly conveyed into the haven of truth.

The third general position, to which I must now turn the reader's attention, is the origin of bilious fever from an epidemic condition, or constitution of the air. As all sound physical knowledge rests on a correct and comprehensive survey of the prominent facts, which have relation to the topic to be elucidated, and as history is philosophy teaching by example, I will advert to Noah Webster's work entitled, "A brief History of Epidemic and Pestilential Diseases," for a species of evidence not easily set aside, and which goes far to obviate the sceptical objections made to the existence of a pestilential state of the atmosphere. Mr. Webster, by his profound investigations, establishes the fact, that bilious fever, as well as influenza, often has its origin in a peculiar noxious state of the air. To his erudite work I must again refer for a more expanded illustration of this remark. My paper merely demands a quotation from that author. Influenza is a disease owing its origin to a wide spreading morbid condition of atmosphere, which is irrespective of its physical mutations. "This epidemic, says he, is evidently the effect of some insensible qualities of the atmosphere; as it spreads with astonishing rapidity over land and sea, *uncontrolled by heat or cold, moisture or drougth.*"—p. 34.

He gives forty-four instances of influenza, occurring in different years from the 1174 down to 1797, from which it is evident, that this epidemic prevailed indiscriminately in cold and hot, in dry and moist, seasons of the year. Sometimes it burst forth from the icy regions of the north, sometimes it commenced its career in Asia or Africa, and at other times it sprang up in the wilds of America.

But I will refer to another malady far more destructive than influenza, which is dependent, as an epidemic disease, on a pestilential constitution of the air. Cholera Morbus is scarcely known in America as an epidemic disease; but it is generally recognized by American physicians as a variety of that congener of bilious affections, seen during summer and autumn throughout the Union. In the East Indies it is known as a fell destroyer of the species. Of this epidemic the best author on the subject, writes thus—"It is proved by the various reports, that the cholera has appeared, and *was equally virulent*, during all states of the atmosphere—amidst all diversity of the circumstances of the people." Again—"The disease has been capable of exerting its influence in every state of the atmosphere, *so far at least as is evident to our senses, or determinable by instruments.*"*

Additional proof is superfluous on this point. Analogy, though neither proof nor illustration, is to be regarded, in contested cases, of some moment, in bringing about a satisfactory decision, provided the case from which the analogy is drawn, bears a manifest resemblance in some one point to the case to be elucidated. This consideration receives full support from the relative aspect that cholera morbus bears towards bilious fever. These diseases are evidently, in our country, the products of similar causes, their treatment is founded on a generally conceded similitude of pathology, and they sustain at times an analogy in the obscurity which envelopes their origin.

In the year 1822, epidemic bilious fever, in its several types, prevailed in Kentucky, Indiana, Virginia, Pennsylvania, and

*Johnson's Medico Chirurgical Review, July, 1825, pp. 134 and 135.

several other states of the Union. In Kentucky and Indiana, there was an unprecedented quantity of rain during the summer of that year. In Pennsylvania and Maryland, there was a considerable drought during the summer. In the Carolina's there was nothing remarkable, and yet they were visited with the fever. Louisiana and Mississippi enjoyed their ordinary seasons, and yet they were extensively assailed by bilious fever. In our county, Jefferson, the most healthy sites were attacked by bilious fever in that fatal summer and autumn. The same fact was observed in the more elevated region constituting the western part of Pennsylvania.

Analogous facts might be adduced from the historical accounts given of epidemic fever, by several eminent authors. But as the design of this short paper is merely to indicate the outlines of this wide territory of indagation, we must terminate the inquiry without any further accession of proof, or protraction of argument.

Before laying down my pen, I will give a part of Dr. Smith's synopsis of the remote causes of infection; comprehending two species of *Koino*, or common miasma.

ORDER II.

INFECTION.

*Genus I.*

Koino Miasma.

*Species* 1.

Protokoino miasma—producing intermittent and remittent fever.

*Species* 2.

Perkoino miasma—producing yellow fever and plague.

The Greek ordinal numeral *protos*, and intensive particle *per*, are prefixed to the word *koino*, to denote, the first the mild and the second the malignant species, of miasmatic effluvium.

Having presented two grounds of contemplation—one narrow and simple and merely confined to the direct physical influences of the atmosphere; the other more large and complicated, comprehending the agency of the whole series of insensible meteorations in the production of bilious fever, I cannot close, without again referring the inquisitive reader to the best writers for information on this highly interesting subject—a subject to which every physician, of the least professional ambition, and philanthropic sensibility, should have all his faculties and energies of mind sensitively alive.

Noah Webster on Pestilence; Dr. E. D. Bancroft on Yellow Fever; Dr. Joseph Mather Smith's Elements of the Etiology and Philosophy of Epidemics; Dr. N. Chapman's eloquent Essay on the Causes, Phenomena, and Laws of Epidemics, with suggestions for their prevention and suppression: which is to be found in the Philadelphia Medical Journal, commencing at p. 352, vol. 8, and running through several numbers; and Ferguson on Marsh Poison—Chapman's Journal, vol. 7,—are books and papers relative to this subject, which may be studied with equal pleasure and profit.

*Louisville, Ky. January*, 1829.

